# Sex-Related Differences in Predictors of Acute Coronary Syndrome in Kosovo: A Cross-Sectional Study

**DOI:** 10.3390/jcm14228002

**Published:** 2025-11-11

**Authors:** Vesa Hoxha, Endrit Dragusha, Shqiponjë Morina, Enera Dragusha, Shpetim Thaqi, Michael Y. Henein, Ibadete Bytyçi

**Affiliations:** 1 Medical Faculty, University of Prishtina, 10000 Prishtina, Kosovo; ve.hoxha@gmail.com (V.H.); i.bytyci@hotmail.com (I.B.); 2 Clinic of Cardiology, University Clinical Centre of Kosovo, 10000 Prishtina, Kosovo; en.dragusha@gmail.com; 3 National Heart and Lung Institute, Imperial College London, London SW7 2AZ, UK; m.henein@imperial.ac.uk; 4 Medical School, University of Nicosia Medical School, Egkomi 2408, Cyprus

**Keywords:** acute coronary syndrome, conventional cardiac risk factors, hypercholesterolemia, uncontrolled diabetes, smoking, older

## Abstract

**Background/Objectives**: Acute coronary syndrome (ACS) in old people is a growing global health problem, with high incidence and mortality. The aim of this study was to assess the cardiovascular risk factors in older patients with ACS, with particular emphasis on sex differences. **Methods**: We retrospectively analyzed 1482 patients with ACS (1184 old patients; men ≥ 55 years and women ≥ 65 years) and 298 young ACS patients (men ≤ 55 years and women ≤ 65 years) from University Clinical Centre of Kosovo. Data on cardiovascular risk factors, echocardiographic, electrocardiographic, angiographic indices and medications were collected from medical records. **Results**: Old ACS patients had higher prevalence of diabetes (50.1 vs. 34.6%; *p* < 0.001), hypertension (79.8 vs. 42.8; *p* < 0.001), multivessel coronary artery disease (88.6 vs. 22.1%; *p* < 0.001) but less prevalent hypercholesterolemia (31.5 vs. 48.2; *p* < 0.001), smoking, family history of coronary artery disease and other noncardiac risk factors compared with young ACS patients (*p* < 0.05, for all). Older women smoked less (26.3 vs. 41.1; *p* < 0.001) and drank less alcohol (0.8 vs. 6.8%; *p* < 0.001) but had higher prevalence of uncontrolled diabetes, arterial hypertension and hypercholesterolemia (*p* < 0.05 for all) compared with older males. Family history for coronary artery disease (CAD) was not significant between groups. Multivariate analysis revealed uncontrolled diabetes (OR = 2.26; 95% CI: 1.104–3.989; *p* < 0.001) and having three or more cardiac risk factors (OR = 3.141; 95% CI: 2.166–4.406; *p* < 0.001) as the strongest independent predictors of ACS in old patients. These associations remained significant when stratified by gender, with even stronger impact in female (uncontrolled diabetes OR = 2.942, 95% CI: 1.644–4.890; *p* < 0.001; ≥3 risk factors OR = 2.821; 95% CI: 1.782–4.436; *p* < 0.001) and in males who smoked (OR: 2.381, 95% CI: 1.109–2.981; *p* < 0.001). **Conclusions**: Uncontrolled diabetes and multiple cardiovascular risk factors are key contributors to ACS in older adults. Early identification and management of these risk factors are essential in reducing the burden of CAD older patients.

## 1. Introduction

Coronary artery disease (CAD) is the most common type of cardiovascular disease worldwide and remains a leading cause of cardiovascular morbidity, mortality, and reduced quality of life [1]. The incidence of CAD is projected to rise further, driven not only by the ongoing trend of population aging but also by cardiovascular risk factors, particularly obesity, diabetes, and metabolic syndrome [2]. Over the past two decades, global population aging has markedly accelerated [3]. The prevalence of CAD varies substantially across demographic and socioeconomic groups, with older adults, men, and certain ethnic populations exhibiting higher risk. Socioeconomic disadvantage, including unemployment, has also been associated with increased CAD burden, although its contribution remains secondary to traditional cardiovascular risk factors such as hypertension, diabetes, and dyslipidemia. Despite a relatively stable prevalence rate, the absolute number of IHD cases continues to rise, primarily driven by global population aging [3,4]. CAD remains a public health concern among older people, emphasizing the need for targeted preventive strategies, effective management of modifiable risk factors, and strengthened health system capacity to address the demands of aging populations [5].

It is well established that lifestyle and social factors significantly influence not only the prevalence but also the clinical course and outcomes of IHD in older adults, playing a central role in shaping gender disparities [6]. Men are more likely to adopt high-risk behaviors such as smoking, excessive alcohol consumption, as well as poor dietary habits and physical inactivity, all of which contribute to accelerated atherosclerosis, adverse CV events, and ultimately increased mortality risk [7,8].

Conversely, women often present with different behavioral and psychosocial risk profiles, including higher levels of stress and depression, which may also negatively impact prognosis [9,10]. Furthermore, socioeconomic factors amplify these disparities, as unequal access to healthcare, education, and resources between men and women impacts disease diagnosis, treatment, and clinical outcomes. Such inequities not only reinforce existing gender gaps but also contribute to worse morbidity and mortality rates in vulnerable populations [11].

Therefore, our study aimed to assess cardiovascular risk factors and sex-related differences in older patients with ACS to enhance understanding of disease characteristics across sexes.

## 2. Methods

### 2.1. Study Design and Patients

This retrospective study analyzed 1482 patients with typical ACS treated across tertiary care hospitals. Patients were stratified by age into two groups: old ACS (men ≥ 55 years and women ≥ 65 years; *n* = 1184) and young ACS (men ≤ 55 years and women ≤ 65 years; *n* = 298) [12,13], recruited from the Clinic of Cardiology, University Clinical Centre of Kosovo, between January 2024 and October 2024. Patients who met the inclusion criteria for ACS including typical or atypical chest pain, electrocardiographic changes suggestive of ischemia, and elevated cardiac biomarkers, were retrospectively analyzed. Significant CAD was defined as ≥70% diameter stenosis in a major epicardial vessel (or ≥50% in the left main). Exclusion criteria included normal coronary angiograms, recent major trauma, acute kidney injury, hepatic failure, or hemorrhagic shock.

The study was conducted in accordance with national and institutional guidelines and the revised Declaration of Helsinki. Ethical approval was obtained from the Institutional Ethics Committee of the University Clinical Centre of Kosovo and the Medical Faculty, University of Prishtina (1489/2025). Patient confidentiality was strictly maintained, and all data were used exclusively for research purposes.

### 2.2. Cardiovascular Risk Factor Assessment

We collected information on CV risk factors based on patients’ medical history at the tertiary care hospital. The data included age, sex, smoking status (classified as current or former smokers, former smoker was defined as an individual who had quit smoking at least six months prior to hospitalization), arterial hypertension (systolic blood pressure ≥ 140 mmHg and/or diastolic blood pressure ≥ 90 mmHg on at least two occasions, or a previous diagnosis of hypertension by a physician/current use of antihypertensive medication) [14], diabetes mellitus (physician-diagnosed diabetes or current use of hypoglycemic agents or insulin) [15], hypercholesterolemia (total cholesterol ≥ 200 mg/dL [5.2 mmol/L] or current use of lipid-lowering therapy) [16], family history of coronary artery disease (first-degree relative with documented coronary artery disease), and alcohol consumption. Uncontrolled diabetes was defined as fasting plasma glucose (FPG) ≥ 7.0 mmol/L or HbA1c ≥ 7.0% despite treatment [17,18]. Among non-cardiac risk factors, we evaluated thyroid disorders and anemia, as well as additional systemic conditions. Kidney dysfunction in our study was defined based on elevated serum creatinine and/or urea levels above the upper limit of the normal reference range, since eGFR data were not available for most patients. This approach was used as a practical surrogate to indicate impaired renal function in the absence of direct eGFR measurements [19]. Peripheral arterial disease (PAD) was defined as a prior clinical diagnosis and/or ankle–brachial index (ABI) < 0.90, or angiographically documented ≥50% stenosis in a major peripheral artery [20]. Carotid artery disease was defined as documented ≥50% carotid stenosis on duplex ultrasound, CT angiography (CTA), or MR angiography (MRA), or a history of prior carotid revascularization [21].

### 2.3. Laboratory and Imaging Assessment

Routine biochemical test results were collected, including complete blood count, fasting blood glucose, cholesterol, triglycerides, albumin, total protein, and kidney and liver function tests.

Echocardiographic data were obtained using standard transthoracic echocardiography performed by experienced cardiologists at the tertiary care hospital, University Clinical Centre of Kosovo. Key echocardiographic parameters included left ventricular end-diastolic diameter (LVEDD), left ventricular end-systolic diameter (LVESD), ejection fraction (EF), and atrial dimensions (right and left atria) [22]. Electrocardiographic (ECG) recordings were analyzed to assess cardiac rhythm and ischemic changes, including T-wave inversion and ST-segment deviations. Information on medications, including angiotensin-converting enzyme inhibitors (ACEIs), angiotensin II receptor blockers (ARBs), beta-blockers, antiplatelet therapy, and lipid-lowering therapy, was obtained from patients’ clinical records.

### 2.4. Statistical Analysis

Categorical variables were expressed as counts with corresponding percentages, while continuous variables were presented as mean ± standard deviation (SD) or, when not normally distributed, as median with interquartile range (IQR). Comparisons of continuous data between groups were performed using the independent two-tailed Student *t*-test, and categorical variables were analyzed with the chi-square test. Associations between categorical and continuous variables were assessed using the chi-square test and point-biserial correlation. Potential predictors of ACS were explored through univariate analyses and subsequently adjusted in multivariate models, both in the overall cohort and stratified by sex. A two-sided *p* value < 0.05 was considered statistically significant. All analyses were carried out using IBM SPSS Statistics, version 26.0 (IBM Corp., Armonk, NY, USA).

## 3. Results

### 3.1. Demographic and Clinical Characteristics of ACS Patients by Age Group

Old patients had more prevalent diabetes (50.1% vs. 34.6%; *p* < 0.001), hypertension (79.8% vs. 42.8%; *p* < 0.001) and multivessel disease (88.6% vs. 22.1%; *p* < 0.001) but less prevalent hypercholesterolemia (31.5% vs. 48.2%; *p* < 0.001), smoking (33.3% vs. 40.5%; *p* < 0.001), family history of CAD (5.1% vs. 10.7%; *p* < 0.001) and other noncardiac risk factors compared with young patients (*p* < 0.05, for all;). Beyond aspirin, clopidogrel was more commonly used in old patients (56.5% vs. 29.8%; *p* < 0.001), while young patients more frequently received prasugrel (56.1% vs. 29.6%; *p* < 0.001). Other medications including ACE/ARBs, diuretics and DOAC/VKA were more frequently used by the older patients (*p* < 0.05 for all). Lipid-lowering therapy was similarly used (*p* = 0.661) by the two groups. ST changes on ECG were more commonly seen in young ACS (73.3% vs. 58.1%, *p* < 0.001), while atrial fibrillation is more frequently reported in older patients (7.4% vs. 2.9%; *p* < 0.05). Previously cardiaovascular events including myocardial infarction, cardiac revascularization, stroke and peripheral artery disease were significantly more frequent in older patients compared to younger patients (*p* < 0.05 for all; Table 1).

### 3.2. Laboratory and Cardiac Imaging Data in Old Versus Young ACS Patients

At admission, older patients had higher fasting blood glucose levels (10.1 ± 6.7 vs. 7.5 ± 4.7 mmol/L; *p* < 0.001), frequently uncontrolled diabetes (63.1% vs. 43.7%; *p* < 0.001), and worse renal function (33.9% vs. 12%; *p* < 0.001) compared to young patients (*p* < 0.005). In contrast, cholesterol levels different differed between groups, with lower total cholesterol (5.1 ± 3.1 vs. 5.9 ± 1.2 mmol/L; *p* = 0.061), LDL-C (2.93 ± 1.6 vs. 3.51 ± 1.3 mmol/L; *p* = 0.041) and HDL-C (1.1 ± 0.9 vs. 1.2 ± 0.7 mmol/L; *p* = 0.556) in the older group compared to the younger group. Triglycerides were not different between groups (2.1 ± 7.1 vs. 2.1 ± 1.4 mmol/L; *p* = 0.881) (Table 2). Older patients had significantly more prevalent three-vessel coronary artery disease (88.6 vs. 22.1%, *p* < 0.001) and less frequently one vessel/two vessel disease and ST segment shift on ECG compared to younger patients (*p* < 0.001; Table 1). Except left atrial (LA) dilatation, a mild reduction in left ventricular (LV) ejection fraction (EF), and slightly increased LV dimensions in older patients (*p* < 0.05), no other significant cardiac structure or function differences were found between groups (*p* > 0.05 for all; Table 1 and Table 2).

### 3.3. Sex Differences in Old ACS Patient Groups

Older male patients had more frequent arterial hypertension (86.1% vs. 74.5%; *p* < 0.001), smoking (41.1% vs. 26.3%; *p* < 0.001) and alcohol use (6.8% vs. 0.8%; *p* < 0.001) while diabetes, uncontrolled diabetes, hypercholesterolemia and number of cardiac risk factors were less prevalent compared to older females (*p* < 0.05 for all). Among non-cardiac risk factors, thyroid disorders, anemia, and liver diseases were more prevalent in women (*p* < 0.05 for all; Table 3). ST shift was more prevalent but T inversion and atrial fibrillation less prevalent in older males compared to older females. Prasugrel was frequently used in old males, while clopidogrel and diuretics were more common used in older females. While previous myocardial infarction and revascularization were more frequent in males compared to females, the prevalence of stroke and peripheral artery disease did not differ (Table 3).

### 3.4. Independent Predictors of ACS in Old Patients

Multivariate analysis revealed uncontrolled diabetes (OR = 2.26; 95% CI: 1.104–3.989; *p* < 0.001) and having three or more cardiac risk factors (OR = 3.141; 95% CI: 2.166–4.406; *p* < 0.001) as the strongest independent predictors of ACS in old patients, followed by age (OR = 1.261; 95% CI 1.019–2.901; *p* = 0.011), hypercholesterolemia (OR = 2.001; 95% CI: 1.437–3.971; *p* = 0.014) and hypertension (OR = 1.121; 95% CI: 1.009–1.741; *p* = 0.033; Table 4).

These associations remained significant when patients were stratified according to sex, with even stronger impact in female (uncontrolled diabetes OR = 2.942; 95% CI: 1.644–4.890; *p* < 0.001; ≥3 risk factors OR = 2.821; 95% CI: 1.782–4.436; *p* < 0.001) and in males who smoked (OR: 2.381; 95% CI: 1.109–2.981; *p* < 0.001; Table 4).

## 4. Discussion

In our cohort, older patients with ACS exhibited distinct risk factor profiles compared with younger individuals. Diabetes, uncontrolled diabetes, and hypertension were more frequent among older patients, whereas smoking and hypercholesterolemia were less common. Within the older subgroup, men more often presented with arterial hypertension, smoking, and alcohol use, while women showed a higher prevalence of diabetes, hypercholesterolemia, and a greater overall burden of cardiovascular risk factors. Notably, uncontrolled diabetes and the presence of three or more risk factors emerged as the strongest correlates of ACS in older adults, particularly among females.

ACS is more common among older individuals, in whom the cumulative effects of cardiovascular risk factors, diabetes, and hypertension lead to a more advanced and often multivessel form of the disease compared with younger patients [23,24]. In older ACS patients, diabetes, especially when poorly controlled and together with hypertension, smoking, and alcohol consumption, was more frequently observed. Such age-related clustering of risk factors highlights that their influence on CAD is not uniform, but rather evolves across different stages of the lifespan. [25,26]. Many researchers have documented the strong genetic contribution to ACS [27,28]. In our study, a positive family history of cardiovascular disease was less frequent among older patients. This observation likely reflects the higher contribution of inherited and shared early-life factors to premature forms of coronary artery disease. However, given the complexity of genetic and environmental interactions, this interpretation should be viewed as descriptive rather than mechanistic. A first-degree relative with CHD increases the risk 1.5–3-fold, and more than tenfold if the event occurs before age 45. Since atherosclerosis develops silently from early adulthood, familial clustering can accelerate progression even without other risk factors, which explains its greater impact in younger patients [29,30]. While observational studies have long established the link between smoking and atherosclerotic cardiovascular disease [31,32,33,34], recently, Levin et al. analyzed data from over 1.5 million participants across multiple datasets to identify genetic loci related to smoking in large Genome-Wide Association Studies (GWASs) of coronary artery disease, stroke, and peripheral artery disease. Their findings suggest that smoking exerts a direct atherogenic effect, with variable impact across different vascular beds [35]. Our findings emphasize the importance of gender-related differences in cardiac risk factors [36]. Although men and women share most traditional risk factors, their relative contribution differs. In younger women, hypercholesterolemia confers a lower risk compared with men. With menopause, however, lipid patterns shift: total cholesterol rises by about 10%, LDL cholesterol by 14%, and lipoprotein(a) by 4–8%, while HDL levels remain stable [37]. Beyond 65 years of age, women have higher mean LDL cholesterol than men [38]. Importantly, evidence from a large Japanese cohort showed that statin therapy provides clear primary prevention benefits for women over 55 with moderately elevated cholesterol levels [39].

Finally, multivariate analysis showed that uncontrolled diabetes and the presence of three or more cardiac risk factors were the strongest independent predictors of ACS in older patients, followed by increasing age and hypercholesterolemia. These associations remained significant after stratification by gender, with diabetes and clustered risk factors exerting a particularly strong influence in women, while smoking emerged as the most important contributor among men.

Uncontrolled diabetes and multiple cardiovascular risk factors are major contributors to ACS in older adults. Diabetes accelerates atherosclerosis, worsens endothelial dysfunction, and heightens vascular inflammation, thereby amplifying ischemic risk. When these processes act in concert with other risk factors, their impact becomes synergistic, leading to higher incidence and severity of coronary artery disease. Early detection and timely management through strict glycemic control, blood pressure and lipid regulation, lifestyle changes, and appropriate pharmacotherapy are essential for reducing future coronary events, with their known impact on cardiac performance and its consequences [40,41].

Study limitations: Our study has certain limitations that merit consideration. Firstly, laboratory data were incomplete, particularly lipoprotein(a) which is an established cardiovascular risk factor that might have provided additional insights. Secondly, the echocardiographic evaluation was not comprehensive. Advanced imaging parameters, including strain, strain rate, and wall motion score index, which offer valuable information on left ventricular intrinsic myocardial function, were not systematically assessed, because of the acute scenario. Another limitation of our study is that patients were not analyzed according to their clinical presentation or the specific type of acute coronary syndrome (STEMI, NSTEMI, or unstable angina). Finally, the retrospective nature of the study is associated with inherent constraints, such as missing data, unmeasured confounders, and the inability to establish causal inferences.

Conclusion: Uncontrolled diabetes and multiple cardiovascular risk factors are key contributors to acute coronary syndrome in older adults. Early identification and management of these risk factors are essential in reducing the burden of coronary artery disease in old patients.

## Figures and Tables

**Table 1:** Demographic and clinical data of patients in older compared to young ACS patients.

Variable	Older ACS	Younger ACS	*p* Value
	(*n* = 1184)	(*n* = 298)	
Cardiac risk factors			
Age (years)	69.4 ± 3.3	51 ± 4.4	<0.001
Male gender (%)	72.7	61.8	0.021
AH (%)	79.8	42.8	<0.001
DM (%)	50.1	34.6	<0.001
Uncontrolled DM (%)	63.1	43.7	<0.001
Hypercholesterolemia (%)	31.5	48.2	<0.001
Smoking (%)	33.3	40.5	0.032
Alcohol use (%)	3.81	5.51	0.147
Family History (%)	5.1	10.7	<0.001
Number of cardiac risk factors			
0 risk factor (%)	9.0	16.2	0.011
1–2 risk factor (%)	73	69.1	0.142
≥3 risk factors (%)	19.9	14	0.043
Other risk factors			
CRF (%)	33.9	12.0	<0.001
Thyroid disorders (%)	3.3	7.1	0.033
CLD (%)	24.9	19.6	0.038
COPD (%)	5.9	1.6	0.044
Anaemia (%)	10	16.8	0.041
Drugs			
LLT (%)	96.4	96.2	0.661
Aspirin (%)	88.6	93.9	0.041
Clopidogrel (%)	56.5	29.8	<0.001
Prasugrel (%)	29.6	56.1	<0.001
DOAC/VKA (%)	12.0	1.10	<0.001
ACE/ARBI (%)	69.2	58.3	<0.001
MRA (%)	11.4	14.6	0.321
Diuretic	51.9	26.8	<0.001
Number of coronaries included			
Single vessel (%)	4.15	32.7	<0.001
Two vessels (%)	7.25	45.2	<0.001
≥3 vessel (%)	88.6	22.1	<0.001
ECG changes			
T inversion (%)	28.0	26.5	0.331
ST depression/elevation (%)	58.1	73.3	<0.001
Previously cardiovascular events			
Myocardial infarction (%)	24.7	4.8	<0.001
PCI/CABG (%)	22.1	3.1	<0.001
Stroke (%)	6.2	0.6	<0.001
PVD (%)	5.9	0.4	<0.001
AF (%)	7.42	2.9	0.041
Complication			
VT (%)	1.3	1.1	0.332
Cardiac arrest (%)	1.1	2.2	0.119

Abbreviations: AF: Atrial fibrillation; AH: Arterial hypertension; CABG: Coronary artery bypass grafting; CLD: Chronic liver disease; COPD: Chronic obstructive pulmonary disease; CRF: Chronic renal failure; DM: Diabetes mellitus; LLT: Lipid-lowering therapy; DOAC: Non–vitamin K antagonist direct oral anticoagulant; PCI: Percutaneous coronary intervention; PVD: Peripheral vascular disease; VKA: Vitamin K antagonist; VT: Ventricular tachycardia.

**Table 2:** Echocardiographic and laboratory indices of patients in older compared young ACS patients.

Variable	Older ACS	Younger ACS	*p* Value
	(*n* = 1184)	(*n* = 298)	
Echocardiographic indices			
LV EDD (mm)	54.3 ± 6.4	48.9 ± 5.9	<0.001
LV ESD (mm)	38.5 ± 7.1	34.1 ± 5.1	<0.001
EF (%)	48.5 ± 9.3	53.4 ± 8.3	0.007
RV (mm)	32.7 ± 7.1	30.2 ± 5.1	0.022
RA transversal (mm)	38.3 ± 6.1	36.4 ± 5.3	0.026
RA longitudinal (mm)	47.2 ± 7.1	43.1 ± 5.1	0.031
LA transversal (mm)	39.1 ± 8.1	36.1± 5.1	0.013
LA longitudinal (mm)	47.4 ± 7.5	38.9± 6.5	<0.001
Laboratory indices			
WBC (10^3^/uL)	10.1 ± 3.3	10 ± 3.3	0.331
RBC (10^6^/uL)	4.4 ± 1.2	4.6± 4.3	0.672
Hgb (g/dL)	13.4 ± 3.3	15.8 ± 4.3	0.041
Hematocrit (%)	39.9 ± 6.3	42.4 ± 6.1	0.022
Platelet (10^3^/uL)	224 ± 73	225 ± 67	0.338
Glycemia (mmol/L)	10.1 ± 6.7	7.6 ± 4.7	<0.001
CRP (mg/L)	31.5 ± 41	20.7 ± 39	<0.001
Urea (mmol/L)	10.4 ± 20	6.48 ± 8.3	0.031
Creatinine (µmol/L)	122 ± 83	101 ± 49	0.022
TSH (mL/UL)	3.1 ± 2.3	2.1 ± 1.4	0.221
Total cholesterol (mmol/L)	5.1 ± 3.1	5.9 ± 1.2	0.061
Triglycerides (mmol/L)	2.1 ± 7.1	2.1 ± 1.4	0.881
LDL-C (mmol/L)	2.93 ± 1.6	3.51 ± 1.3	0.041
HDL-C (mmol/L)	1.1 ± 0.9	1.2 ± 0.7	0.556

Abbreviations: EDD: End diastolic diameter; ESD: End systolic diameter Hgb: Hemoglobin; HDL-C: High-density lipoprotein cholesterol; LA: Left atrium; LDL-C: Low-density lipoprotein cholesterol; LV: Left ventricle; RA: Right atrium; RBC: Red blood cell; RV: Right ventricle; TSH: Thyroid-stimulating hormone; WBC: White blood cell.

**Table 3:** Demographic and clinical indices in older compared to young ACS patients by gender.

Variable	Older ACS	Older ACS	*p* Value
	(Male; *n* = 861)	(Female; *n* = 323)	
Cardiac risk factors			
Age (years)	68.4 ± 3.3	70.4 ± 3.3	0.091
AH (%)	86.1	74.5	<0.001
DM (*n*, %)	43.1	58.3	0.012
Uncontrolled DM	57.4	69.7	<0.001
Hypercholesterolemia (%)	28.2	35.9	<0.001
Smoking (%)	41.1	26.3	<0.001
Alcohol (%)	6.8	0.8	<0.001
Family History (%)	5.3	5.0	0.982
Number of cardiac risk factors			
0 risk factor (%)	7.8	10	0.113
1–2 risk factor (%)	74.1	72.4	0.311
≥3 risk factors (%)	14.9	25	<0.001
Other risk factors			
CRF	34.6	32.8	0.123
Thyroid disorders	1.5	5.3	0.014
CLD	28.6	19.9	<0.001
COPD	4.9	6.6	0.147
Anaemia	4.7	16.1	<0.001
Drugs			
LLT (%)	97.1	96.0	0.572
Aspirin (%)	88.2	89.1	0.623
Clopidogrel (%)	50.1	63.8	<0.001
Prasugrel (%)	39.4	19.7	<0.001
NOAC/VKA	12.2	11.8	0.141
ACE/ARBI (%)	68.4	69.9	0.349
MRA (%)	10.3	12.7	0.126
Diuretic	45.8	58.1	<0.001
Number of coronaries included			
Single vessel (%)	1.2	7	<0.001
Two vessels (%)	6.3	8.2	0.091
≥3 vessel (%)	92.5	84.8	0.032
ECG changes			
T inversion	22.2	30.0	<0.001
ST depression/elevation	63.7	52.4	<0.001
Previously cardiac events			
Myocardial infarction (%)	27.9	21.5	0.044
PCI/CABG (%)	25.3	19.1	0.042
Stroke (%)	5.9	6.4	0.446
PVD (%)	5.2	6.5	0.121
AF	6.1	11	0.012
Complication			
VT	1.3	1.2	0.249
Cardiac arrest	1.1	1.1	0.181

Abbreviations: AF: Atrial fibrillation; AH: Arterial hypertension; CABG: Coronary artery bypass grafting; CLD: Chronic liver disease; COPD: Chronic obstructive pulmonary disease; CRF: Chronic renal failure; DM: Diabetes mellitus; LLT: Lipid-lowering therapy; NOAC: Non–vitamin K antagonist oral anticoagulant; PCI: Percutaneous coronary intervention; PVD: Peripheral vascular disease; VKA: Vitamin K antagonist; VT: Ventricular tachycardia.

**Table 4:** Predictors of older ACS.

Variable	Univariate Predictors	*p*	Multivariate Predictors	*p*
	OR (95% CI)	Value	OR (95% CI)	Value
All patients				
Age	1.489 (1.032–2.515)	<0.001	1.261 (1.019–2.901)	0.011
Female gender	0.827 (0.278–3.999)	0.033	0.771 (0.232–1.089)	0.462
Uncontrolled diabetes	2.489 (1.302–5.101)	<0.001	2.267 (1.104–3.989)	<0.001
Arterial hypertension	1.410 (1.132–2.556)	0.008	1.121 (1.009–1.741)	0.033
Hypercholesterolemia	1.188 (1.045–2.311)	<0.001	2.001 (1.437–3.971)	0.014
Smoking	1.573 (1.359–1.921)	<0.001	1.532 (1.311–2.821)	0.011
Family history	0.733 (0.035–1.909)	0.22		
≥3 risk factors	3.674 (1.911–5.489)	<0.001	3.141 (2.166–4.406)	<0.001
Male patients of older ACS				
Age	1.289 (0.932–3.515)	0.33		
Uncontrolled diabetes	1.507 (1.197–2.864)	<0.001	1.181 (1.092–2.951)	0.002
Arterial hypertension	1.660 (1.232–2.756)	0.001	1.155 (1.031–1.892)	0.042
Hypercholesterolemia	1.276 (1.005–2.433)	0.01	1.276 (0.905–2.433)	0.093
Smoking	1.913 (1.436–2.548)	<0.001	2.381 (1.109–2.981)	<0.001
Family history	0.222 (0.015–1.305)	0.33		
≥3 risk factors	2.663 (1.478–4.919)	<0.001	2.608 (1.465–3.893)	<0.001
Female patients of older ACS				
Age	1.346 (0.221–3.541)	0.47		
Uncontrolled diabetes	2.242 (1.201–3.499)	<0.001	2.942 (1.644–4.890)	<0.001
Arterial hypertension	1.567 (1.028–2.965)	0.01	1.682 (1.101–2.809)	0.021
Hypercholesterolemia	1.802 (1.112–3.399)	<0.001	2.873 (2.106–4.022)	<0.001
Smoking	1.113 (1.036–1.948)	0.01	1.587 (0.801–2.121)	0.094
Family history	0.989 (0.312–1.124)	0.36		
≥3 risk factors	2.657 (1.121–3.901)	<0.001	2.821 (1.782–4.436)	<0.001

## Data Availability

Data may be made available by the corresponding author upon reasonable request and with approval from the institutional ethics committee.
